# Micropulse transscleral cyclophotocoagulation in the treatment of autosomal recessive bestrophinopathy combined with angle closure glaucoma: a case report

**DOI:** 10.3389/fmed.2025.1567964

**Published:** 2025-08-08

**Authors:** Jinkun Liu, Yingying Xue, Weiyi Huang, Yuhong Wang

**Affiliations:** Xiamen Eye Center and Eye Institute of Xiamen University, School of Medicine, Xiamen Clinical Research Center for Eye Diseases, Xiamen Key Laboratory of Ophthalmology, Fujian Key Laboratory of Corneal and Ocular Surface Diseases, Xiamen Key Laboratory of Corneal and Ocular Surface Diseases, Translational Medicine Institute of Xiamen Eye Center of Xiamen University, Xiamen, Fujian, China

**Keywords:** autosomal recessive bestrophinopathy, angle closure glaucoma, micropulse transscleral cyclophotocoagulation, vitreous body, intraocular pressure

## Abstract

**Background:**

Autosomal recessive bestrophinopathy (ARB) comprises remarkable retinal dystrophy characterized by yellowish subretinal lesions scattered in the posterior pole and is always accompanied with refractory angle-closure glaucoma (ACG). The treatment of ACG patients with ARB is a major challenge for all ophthalmologists.

**Case presentation:**

A 12-year-old female child was diagnosed with ARB and ACG and presented with discrete, round, yellow–white deposits of variable sizes scattered in the retina, retinoschisis in the macular, shallow anterior chamber depth and angle closure with uncontrolled intraocular pressure (IOP). Micropulse transscleral cyclophotocoagulation (MP-TCP) successfully deepened anterior chamber, lowered IOP and resolved retinoschisis. However, the postoperative deepening of the anterior chamber began to regress 12 days after surgery and stabilized 142 days after surgery, the retinoschisis reoccurred 67 days after surgery.

**Conclusion:**

This case revealed that changes in vitreous condition may play an important role in the formation of retinoschisis. MP-TCP, which induces vitreous compression and increases osmotic pressure on the retina, could be used to treat young ACG patients with ARB to avoid other complicated surgeries and vision-threatening postoperative complications. However, the theory needs to be confirmed by further studies.

## Background

Autosomal recessive bestrophinopathy (ARB) is a distinct inherited retinal dystrophy characterized by multifocal yellowish subretinal lesions scattered in the posterior pole, retinal pigment epithelium (RPE) irregularities, intraretinal fluid and often associated with hyperopia and shallow anterior chamber. ARB, first described by Burgess et al. in 2008, is caused by biallelic BEST1 pathogenic variants (1). Mutations in BEST1 may result in defects during ocular growth and later-onset retinal dystrophy (2, 3). Angle-closure glaucoma has been shown to affect approximately 50% of those with ARB (4). Owing to its early onset age and abnormal choroid and retina, angle closure glaucoma with ARB presents a major challenge to glaucoma surgeons. Both filtration surgery and cataract surgery carry a high risk of malignant glaucoma postoperatively (5, 6). Here, we report an ARB with angle closure glaucoma case in which ACG and retinoschisis were successfully managed with a simple treatment regimen.

## Case presentation

A 12-year-old female presented with progressive bilateral blurred vision for 2 months. She initially visited a local hospital, where her intraocular pressure (IOP) was 31.7 mmHg and 42.8 mmHg in the right and left eyes, respectively. She was prescribed pilocarpine, timolol, travoprost eye drops and oral methazolamide; however, her IOP remained in the 30’s and her vision continued to decline.

**Figure 1:**
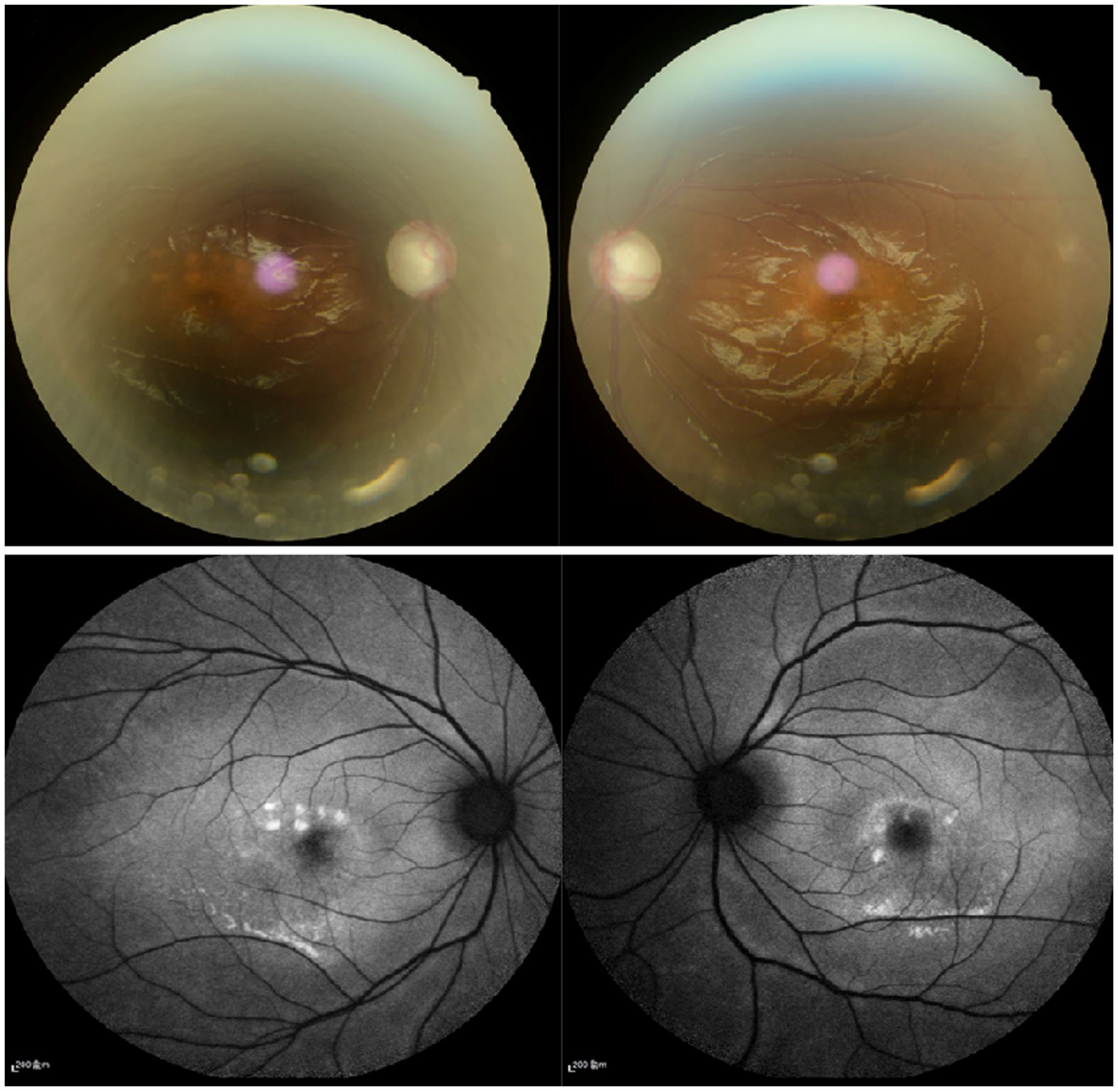
**(A)** Fundus examination show that the cup-to-disc ration were 0.9 in both eyes. **(B)** Fundus autofluorescence revealed that discrete, round deposits of variable sizes scattered in the retina between the upper and lower vascular arches.

**Figure 2:**
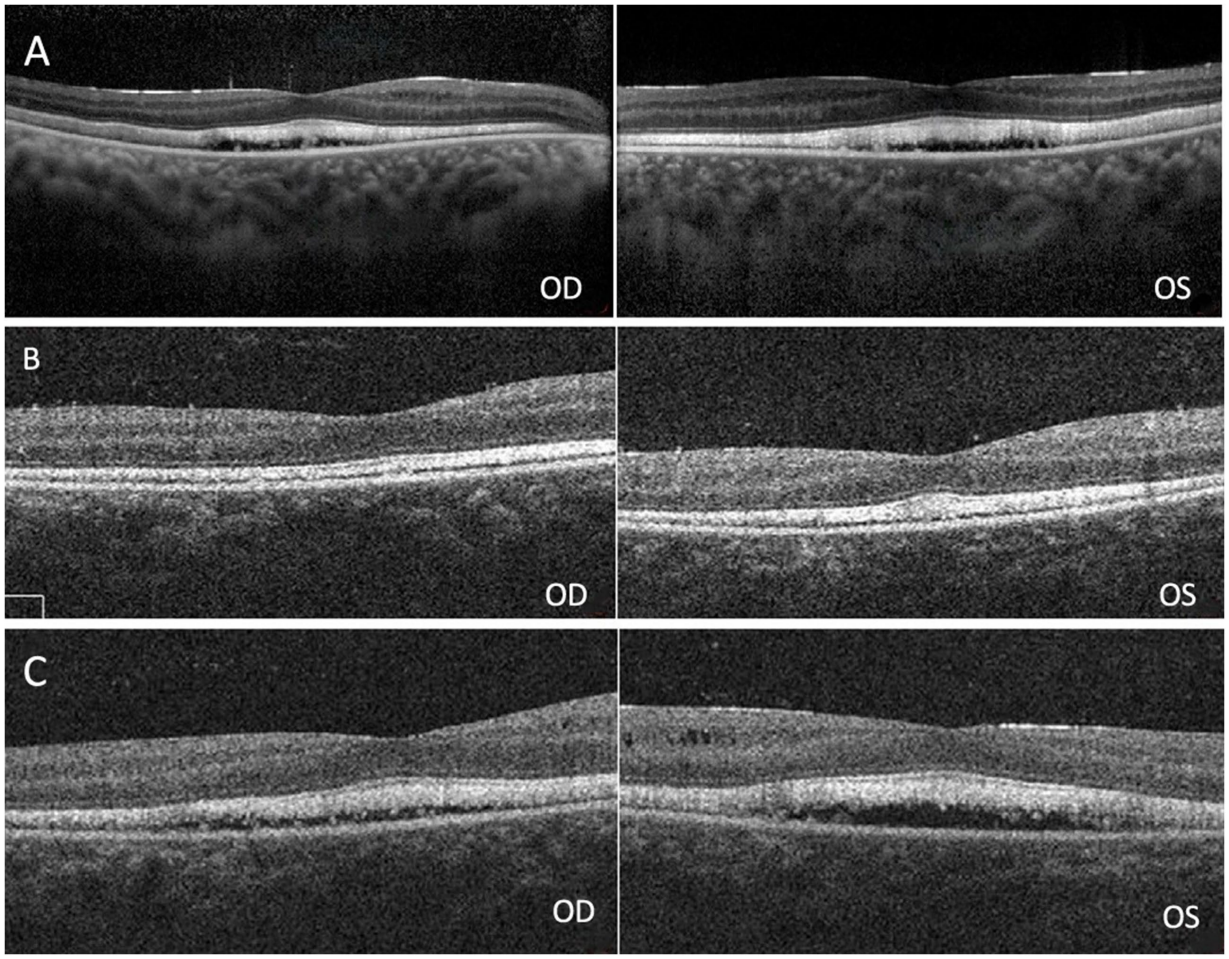
**(A)** Spectral domain optical coherence tomography (SD-OCT) revealed macular edema in both eyes. **(B)** Retinoschisis resolved completely in both eyes 12 days after surgery. **(C)** Macular edema recurred 67 days after surgery.

**Figure 3:**
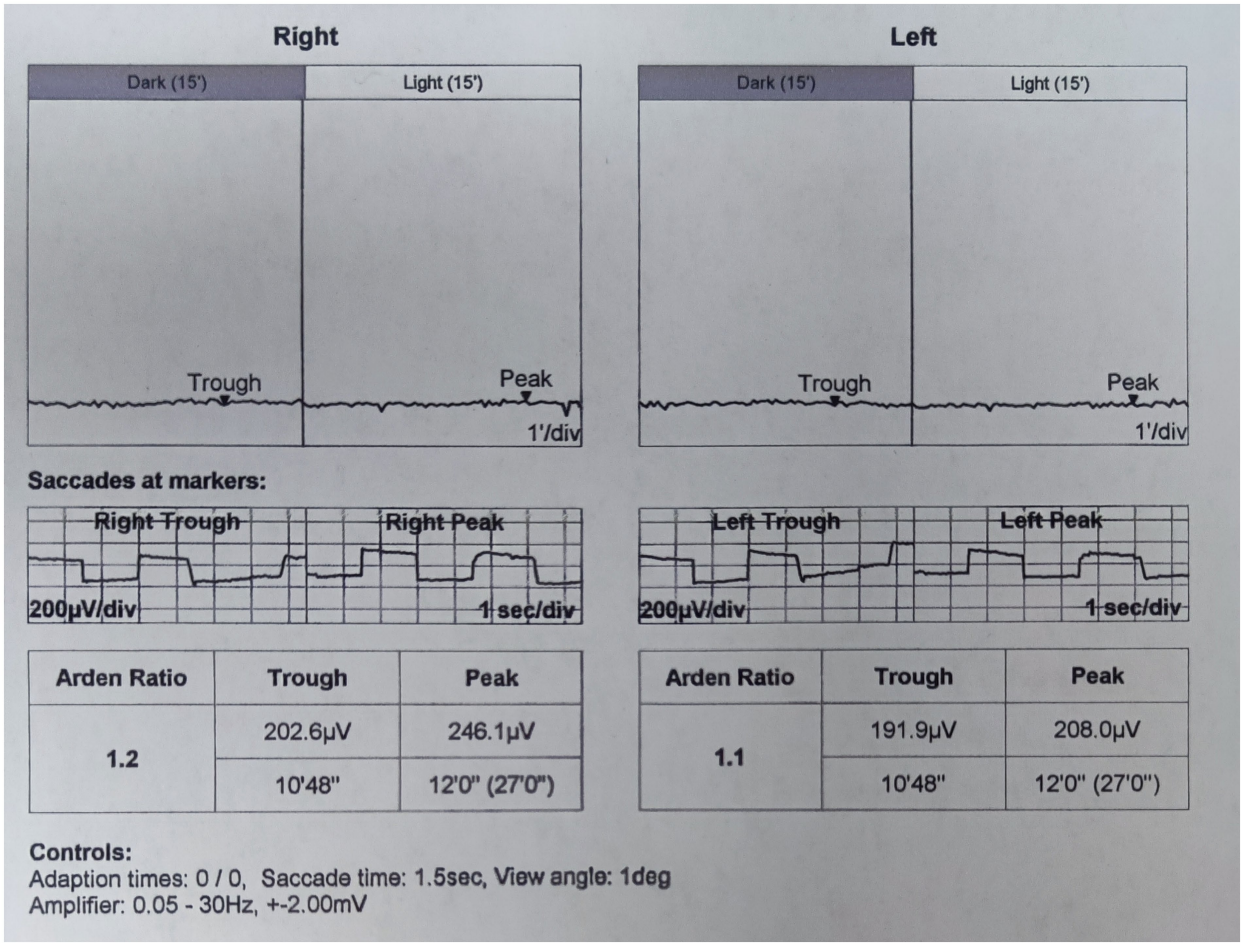
EOG revealed a decreased light rise-to-dark trough ration in both eyes.

On presentation, her best corrected visual acuity (BCVA) was 0.4 in the right eye, and count fingers at 40 cm in the left eye. Her IOP was 24.1 mmHg in the right eye and 37.2 mmHg in the left eye. On further questioning, her guardian denied history of eye diseases in her family and no history of systemic diseases or any recent drug use. The slit-lamp examination revealed a shallow anterior chamber both centrally and peripherally; the pupil diameter was approximately 2 mm in both eyes, and the direct reaction and consensual reaction were slow for the use of pilocarpine, the lenses were clear. Funds examination and fundus autofluorescence (FAF) revealed that the cup-to-disc ratio were 0.9 in both eyes and discrete, round, yellow–white deposits of variable sizes scattered in the retina between the upper and lower vascular arches (Figure 1). Static and dynamic gonioscopy demonstrated circumferential grade IV angle by Scheie grading both eyes. Ultrasound biomicroscopy (UBM) revealed angle closure and anterior rooting of the iris. The aqueous depth (AD, measured from the corneal endothelium to the anterior surface of the crystalline lens or intraocular lens) was 1.94 mm in the right eye and 1.80 mm in the left eye, and the axial length (AL) was 21.41 mm in the right eye and 21.30 mm in the left eye when measured using IOL-Master (HAAG-STREIT AG, Switzerland). The lens thickness (LT) was 3.93 mm in the right eye and 3.97 mm in the left eye. Spectral domain optical coherence tomography (SD-OCT) revealed macular edema, and the subfoveal choroid thickness was 481 μm in the right eye and 448 μm in the left eye (Figure 2A), thicker than the previously reported range in the Chinese population (7). Electrooculography (EOG) was performed according to the current International Society for Clinical Electrophysiology of Vision (ISCEV) standards and revealed characteristic absent light rise, a decreased light rise-to-dark trough ratio (LP: DT), a value of 1.2 in the right eye and 1.1 in the left eye (normal between 1.8–2.5), suggestive of the best disease (Figure 3). Whole-exome sequencing of the patient identified compound heterozygous mutations in BEST1, NM_004183: exon5: c.604C>T: p. Arg202Trp, NM_004183: c.867+97G>A. Further Sanger sequencing of the parents confirmed that both the father and the mother had a heterozygous mutation (farther: BEST1, NM_004183: exon5: c.604C>T: p. Arg202Trp; mother: BEST1, NM_004183: c.867+97G>A). The inheritance pattern was in accordance with ARB.

Based on previous experience, even lens extraction may fail in such cases (5). MP-TCP is a modified application of continuous wave transscleral cyclophotocoagulation (CW-TCP). The Iridex Cyclo G6 Glaucoma Laser System (Iridex, Mountaion View, CA) and Micropulse P3 probe was used with a power of 2000 mw and a duty cycle of 31.3% to perform the treatment. MP-TCP treatment was performed by placing the Micropulse P3 probe at the limbus and perpendicular to the surface of the globe. The probe was then moved in a continuous sweep, sliding motion over each quadrant, taking 10 s for each sweep, and laser treatment was not delivered to the 3 and 9 o’clock positions. The overall treatment time was 200 s for both eyes.

**Figure 4:**
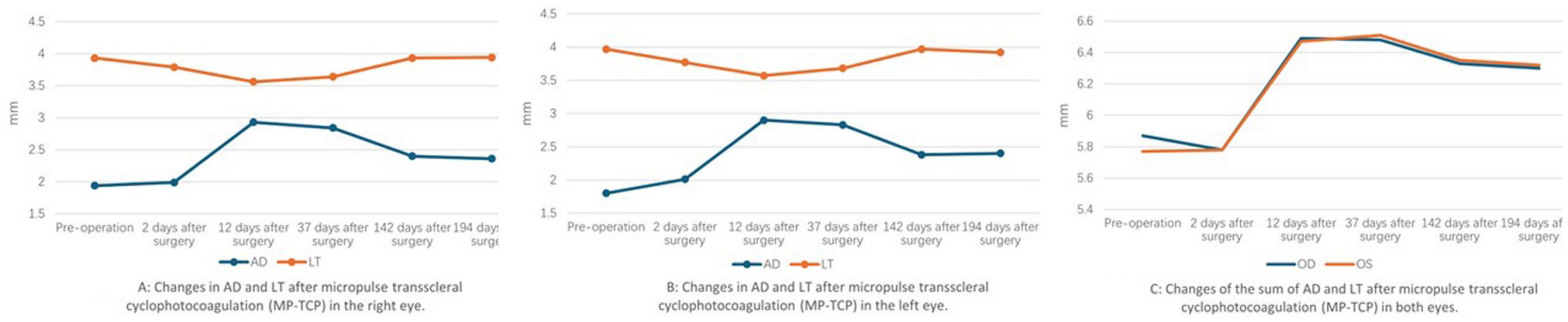
Changes of AD and LT after micropulse transscleral cyclophotocoagulation (MP-TCP) in both eyes.

Two days after the laser, the IOP decreased to 15.3 mmHg in the right eye and 14.9 mmHg in the left eye. The AD was 1.99 mm in the right eye and 2.01 mm in the left eye, as measured by the IOL-Master. The LT was 3.79 mm in the right eye and 3.77 mm in the left eye. By postoperative day 12, the IOP had decreased to 11.1 mmHg in the right eye and 14.4 mmHg in the left eye. The AD deepened significantly from 1.94 mm to 2.93 mm in the right eye and 1.80 mm to 2.90 mm in the left eye, the LT decreased to 3.56 mm in the right eye and 3.57 mm in the left eye. The retinoschisis resolved completely in both eyes (Figure 2B). Thirty-seven days after surgery, the IOP was 18 mmHg in the right eye and 17.3 mmHg in the left eye, the AD was 2.84 mm in the right eye and 2.83 mm in the left eye, the LT was 3.64 mm in the right eye and 3.68 mm in the left eye. Sixty-seven days after surgery, OCT showed macular edema in both eyes (Figure 2C). By postoperative day 142, the AD was 2.4 mm in the right eye and 2.38 mm in the left eye, the LT was 3.93 mm in the right eye and 3.97 mm in the left eye. 194 days after surgery, the IOP was 17.3 mmHg in the right eye and 19.8 mmHg in the left eye without any antiglaucoma medications. The AD was 2.36 mm in the right eye and 2.40 mm in the left eye, the LT was 3.94 mm in the right eye and 3.92 mm in the left eye (Figure 4), the macular edema was detected by OCT in both eyes. 422 days after surgery, the IOP was 18.3 mmHg in the right eye and 20.6 mmHg in the left eye without any antiglaucoma medications, the BCVA was 0.4 in the right eye and 0.05 in the left eye.

## Discussion and conclusions

Here we report an ARB with angle closure glaucoma who successfully managed ACG and retinoschisis with MP-TCP. Bestrophinopathies are a spectrum of inherited retinal dystrophies caused by pathogenic variation in the Bestrophin 1 protein, which is encoded by the BEST 1 gene (8). In addition to ARB, the other bestrophinopathies are Best vitelliform macular dystrophy (BVMD), adult-onset vitelliform macular dystrophy and autosomal dominant vitreretinochoroidopathy. Over 100 different BEST1 mutations have been described in families affected by BEST disease (1, 9). Mutations in BEST1 may result in defects during ocular growth and later-onset retinal dystrophy. In addition, transcription factors such as microphthalmia-associated transcription factor (MITF), orthodenticle homeobox 2 (OTX2) and cone–rod homeobox (CRX) may regulate the transcription of BEST1 and when mutated, they may affect BEST1 expression, which can lead to changes in the development of RPE and anterior segment malformations in the human eye (2, 3). Both mutations of the patient have been reported previously, Li’s research proved that the c.867+97G>A deep intronic variant is a common founder variant for Chinese patients with ARB. Furthermore, they also proved that the deep intronic variant c.867+97G>A had two splicing or transcript products, one is the normal splicing products and the other contained a 203-nt intron retention which generated a premature termination codon downstream (10, 11).

Treatment of ACG in ARB patients is difficult, up to 50% of ARB patients are at risk of angle closure glaucoma. Compared with primary angle closure glaucoma (PACG) patients, patients with ARB are younger and have abnormal ciliary body morphology and high posterior pressure due to choroidal thickening in combination with liquefied vitreous. Yttrium-aluminum-garnet (YAG) laser peripheral iridotomy is not sufficient to lower IOP, though lens extraction in ACG can relieve pupillary block and angle crowing and has become the first-line treatment for PAC patients with or without coexisting cataracts (12). Lens extraction is also not effective in controlling IOP because it cannot open the anterior chamber angle to a sufficient degree and even usually leads to postoperative malignant glaucoma due to the increased pressure gradient between the anterior and posterior segments (5). Filtering surgery is also not recommended for ARB patients because of its complex complications (6).

CW-TCP lowers IOP by directly damaging the ciliary body, the site of aqueous fluid production. As a result of continuous high intensity energy, adverse effects, such as postoperative pain and intraocular inflammation, especially hypotony, limit its use to eyes with limited visual acuity. In contrast to conventional laser delivery, MP-TCP uses a diode laser to deliver a controllable pulsed laser with rest periods between pulses to the ciliary body epithelium and stroma, thus reducing intraocular pressure (IOP). The rest periods allow nonpigmented adjacent tissues to stay below their thermal coagulation threshold, thus minimizing potential complications (13). Satisfactory results have been reported with MP-TCP for refractory glaucoma in adults (13, 14). Multiple studies have assessed the role of MP-TCP in the treatment of refractive glaucoma in pediatric patients, but the conclusions are controversial (15, 16). The 12-year-old patient described here showed good results. The AD gradually deepened and reached its deepest depth at 12 days after surgery; meanwhile, the LT became thinnest at the same time. By 142 days after surgery, the AD and LT became stable, and the LT was nearly the same as it was preoperatively; however, the AD was significantly deeper than it was preoperatively (Figure 4). The mechanism is likely that the MP-TCP shrinks the ciliary body, inducing posterior ciliary body rotation and then straightening the lens zonules. These changes lead to changes in AD and LT, because of the low energy dose and greater regenerative ability of the ciliary body (16), these changes gradually weakened and stabilized at 142 days after surgery.

MP-TCP leads not only to a deeper anterior chamber and an obvious reduction in IOP but also the resolution of retinoschisis. The traditional view of retinoschisis in ARB is that it is secondary to RPE dysfunction (17). However, the change of retinoschisis in this patient implied that RPE dysfunction was not the only reason for retinoschisis. Shi cured a 26-year-old patient diagnosed with ARB and ACG using low dose transscleral cyclophotocoagulation after a failure surgery of phacoemulsification, intraocular lens implantation and goniosynechialysis. A significantly deepened AD was observed until 6 months following TCP treatment, and there was no recurrence of retinoschisis. The authors concluded that the vitreous plays a pathogenic role in the formation of retinoschisis and ACG and that TCP can induce vitreous liquefication, thus decreasing vitreous traction to the macular and resolving macular retinoschisis (5). However, in this patient, retinoschisis recurred at 67 days and remained stable after MP-TCP; moreover, the changes in AD and LT decreased. This phenomenon reminds us that there are other mechanisms for the phenomenon. The vitreous condition may play a significant role in the pathogenesis of retinoschisis. The combined anterior chamber depth and lens thickness increased significantly at 12 days after surgery (Figure 4), which means that the vitreous was compressed significantly; accordingly, the increased osmotic pressure on the retina induced the disappearance of macular edema; however, 37 days after surgery, the vitreous volume began to recover, the osmotic pressure on the retina began to decline gradually, and retinoschisis recurred at 67 days. However, the theory needs to be confirmed by further experimental studies.

This report proposes a novel treatment for ACG patients with ARB; more importantly, it improves our understanding of the role of the vitreous body in the pathogenesis of ACG patients with ARB and the mechanisms of MP-TCP in the treatment of ARB. Although more studies are needed to further explain the pathogenesis of ARB, this case suggests that MP-TCP could be used to treat patients with ARB and other kinds of ACG to avoid postoperative complications.

## Data Availability

The datasets presented in this study can be found in online repositories. The names of the repository/repositories and accession number(s) can be found in the article/supplementary material.
